# Geographical variation analysis of all-cause hospital readmission cases in Winnipeg, Canada

**DOI:** 10.1186/s12913-015-0807-2

**Published:** 2015-04-01

**Authors:** Yang Cui, Mahmoud Torabi, Evelyn L Forget, Colleen Metge, Xibiao Ye, Michael Moffatt, Luis Oppenheimer

**Affiliations:** Evaluation Platform, The George and Fay Yee Centre for Healthcare Innovation, 200-1155 Concordia Avenue, Winnipeg, Manitoba R2K 2M9 Canada; Winnipeg Regional Health Authority, 200-1155 Concordia Avenue, Winnipeg, Manitoba R2K 2M9 Canada; Department of Community Health Sciences, Faculty of Medicine, University of Manitoba, Winnipeg, Manitoba R3E 0 W1 Canada; Departments of Surgery & Family Medicine, Faculty of Medicine, University of Manitoba, Winnipeg, Manitoba R3E 0 W1 Canada; Manitoba Health, 300 Carlton Street, Winnipeg, Manitoba R3B 3 M9 Canada

**Keywords:** Hospital readmissions, Bayesian disease mapping, Relative risk, Spatial pattern

## Abstract

**Background:**

Hospital readmission is costly and potentially avoidable. The concept of virtual wards as a new model of care is intended to reduce hospital readmissions by providing short-term transitional care to high-risk and complex patients in the community. In order to provide information regarding the development of virtual wards in the Winnipeg Health Region, Canada, this study used spatial statistics to identify geographic variations of hospital readmissions in 25 neighborhood clusters.

**Methods:**

The data were obtained from the Population Health Research Data Repository housed at the Manitoba Centre for Health Policy. We used a Bayesian Disease Mapping approach which applied Markov chain Monte Carlo (MCMC) for cluster detection.

**Results:**

Between 2005/06 and 2008/09, 123,842 patients were hospitalized in all Winnipeg hospitals. Of these, 41,551 (33%) were readmitted to hospital in the year following discharge. Most of these readmitted patients (89.4%) had 1–2 readmissions, while 11.6% of readmitted patients had more than 2 readmissions after initial discharge. The smoothed age- and sex- adjusted relative risk rates of hospital readmission in 25 Winnipeg neighborhood clusters ranged between 0.73 and 1.27. We found that there were spatial cluster variations of hospital readmission across the Winnipeg Health Region. Seven neighborhood clusters are more likely to be significant potential clusters for hospital readmissions (*p* < .05), while six neighborhood clusters are less likely to be significant potential clusters.

**Conclusions:**

This study provides the foundation and implementation guide for the Winnipeg Regional Health Authority virtual ward program. The findings will also help to improve long-term condition management in community settings and will help program planners to assure the efficient use of healthcare resources.

## Background

Hospital readmission is common and costly for any healthcare system. In Canada, it has been estimated that the costs of readmissions are $1.8 billion Canadian dollars in 2010 and an estimated $10,404 per individual hospitalization [1]. Some evidences has shown that between 9% and 59% of readmissions were potentially avoidable by improving care before and after discharge [2]. Reducing avoidable hospital readmissions is key to improving efficiency and patient health outcomes across a healthcare system [3,4].

In order to reduce hospital readmission and costs, an intervention program known as “virtual wards” was initially introduced by Geraint Lewis in the United Kingdom (UK) [5]. The function of virtual wards is to reduce hospital readmissions by providing short-term transitional care from the hospital to the home to high-risk and complex patients in the community [6]. The virtual ward usually provides multispecialty case management and preventive care at the patient’s home in the community. The goal of a virtual ward intervention is to improve the integration of health care for patients at high risk of future hospitalization. The use of the virtual ward is an appropriate form of home-based care for persons who are considered to be at high risk for future admission or readmission to hospitals.

There has been growing interest in virtual wards in Canada due to issues related to continuity of care after discharge and high costs associated with hospital readmissions. Currently, there are three virtual ward programs in Canada [5]. Among these three virtual ward programs, a pilot study pursuing the establishment of virtual ward services is underway in the Northeast and West sectors of the Winnipeg Health region. These sectors comprise four community (administrative) areas or about 33% of Winnipeg’s population. Persons with a history of readmissions to hospital or long stay have been selected for complex case management at home. One of the goals of developing virtual wards for these four geographic areas is to assist patients after discharge from hospital to identify clinical problems before they become serious and to improve the quality and efficiency of healthcare services for individuals at high risk for (re-)hospitalization. Interdisciplinary teams including primary care physicians, nurse practitioners, dieticians, pharmacists, occupational therapists, social workers, case coordinators, clinical resource nurses, multi-skilled workers and other services in the community area (e.g. community mental health) agree on referrals to the virtual ward services (now called hospital home teams or HHTs), elicit the patient’s primary goals and then determine how best to meet these goals. Regular meetings amongst team members and provision of care in the home are key to reducing readmissions to hospital. A project manager and measurement specialist continually monitor the HHTs population, needs and service provision. The HHT service is now seen as intensive primary care offering “intensive case management which leverages existing resources”. An evaluation of HHTs is pending.

In order to inform planning regarding the development of HHTs for the entire Winnipeg Health Region, it was important to identify the geographic distributions of persons at risk for hospital readmission. Winnipeg is the capital of Manitoba, Canada, and has a population of 730,018 according to the Canada 2011 census [7]. It is comprised of 25 geographically defined neighborhood clusters for the purpose of planning and delivering health services, each with a population of approximately 27,000 people. The Winnipeg Health Region provides health services to meet a variety of needs for the population in these areas. Neighborhood clusters were defined by the Winnipeg Regional Health Authority and the City's Community Services department in partnership with associated community groups. The Clusters follow neighborhood boundaries and are defined based on population and natural community boundaries. The neighborhood clusters are grouped together to make up the larger community areas. Detailed analysis and comparison of health and social information is possible at the neighborhood cluster level. The population health status and healthcare service needs among 25 neighborhood clusters differ [8].

In this paper, we: (1) identify the demographic, clinical and healthcare utilization characteristics of the targeted population needing HHT services; and, (2) examine the spatial cluster of patients at risk to hospital readmission in the Winnipeg’s 12 community areas comprised of 25 neighborhood clusters.

## Methods

### Data source and study measurements

Since this study is a secondary analysis using the population-based administrative health data, no written informed consent for participant in the study was obtained. Data were obtained from the Population Health Research Data Repository (PHRDR) housed at the Manitoba Centre for Health Policy, University of Manitoba. The PHRDR is a de-identified data repository and holds records for virtually all contacts with the provincial healthcare system, including physicians, hospitals, personal care homes, home care, and pharmaceutical prescriptions of all registered individuals [9].The data repository provides a comprehensive historical collection of administrative, registry, survey and other data on all residents of Manitoba. Permission for use of the PHRDR was sought and granted from the MCHP. A research agreement was signed between the MCHP and the Principal Investigator of this study to ensure confidentiality, privacy and consistency with the approved study protocol are followed by all team members. We used the hospital abstracts databases to capture data on all records of hospital admission for residents in the Winnipeg Health Region. The hospital abstracts database contains patients’ age, gender, length of (hospital) stay, diagnosis, emergent admission and discharge. In this study, the clinical and healthcare utilization characteristics such as Charlson Comorbidity Index (CCI), having a family doctor and having an emergent admission, were obtained from the hospital abstracts. CCI contains 19 categories of comorbidity, which are primarily defined using ICD-10-CM diagnoses codes. The CCI was used to measure the patient’s overall comorbidity at the initial hospitalization; the higher the score, the more severe the burden of comorbidity [10]. Information about patient’s marital status and household income based on neighborhood of residence were obtained from Health Registry and census databases. A quintile split of total family income was used to create five socioeconomic groups ranging from most disadvantaged (quintile 1) to most advantaged (quintile 5).

A hospital readmission was defined as a hospitalization within one year of a previous discharge from hospital. Patients who have at least one readmission to hospital after a discharge were included. Hospital readmissions related to pregnancy, childbirth, abortion were excluded from the analysis. All deaths in hospital during the index admission were also excluded. Hospital discharges of persons resident in Winnipeg from hospitals in the Winnipeg Health Region occurring in four fiscal (April 1 to March 31st) years 2005/06 and 2008/09 were examined.

### Study subjects

The study population included all patients who were age 40 and older living in the Winnipeg Health Region and had continuous healthcare coverage by the Manitoba Health. Manitoba Health is the provincial government providing comprehensive universal health insurance to all residents in Manitoba. The study population was selected from hospital abstracts database dating from April 1, 2005-March 31, 2006 to April 1, 2008-March 31, 2009.

### Statistical analysis

Patients were geocoded to 25 neighborhood clusters in the city of Winnipeg using 6-digit postal codes which were on patients’ health records. These 25 neighborhood clusters were numbered 1, 2, …--, 25 and are the geographic units used in the spatial model.

To visualize spatial variation in hospital readmission, a Bayesian disease mapping (BYM) approach was used to detect the relative risk rates of 25 Winnipeg neighborhood clusters. First, we calculated the number of cases of readmission and the number of expected cases of readmission for each neighborhood cluster, adjusted by age group quartiles (40–51, 52–62, 63–74, 75+) and gender (male, female). Second, we used a Bayesian approach which applied Markov chain Monte Carlo (MCMC) for spatial pattern detection [11-14]. This approach was first used by Besag et al. [11] and the model consists of two parts. In the first part, the cases are assumed to follow a Poisson distribution with an area specific parameter

$$ {\theta}_{\mathrm{i}}\kern0.5em {\mathrm{E}}_{\mathrm{i}} $$

$$ {\mathsf{C}}_{\mathsf{i}}\sim \mathsf{Poisso}\mathrm{n}\kern0.5em \left({\theta}_{\mathsf{i}}\kern0.5em {\mathsf{E}}_{\mathsf{i}}\right), $$

where C _i_ and E _i_ are the observed and expected number of hospital readmission in neighborhood cluster i, respectively. The second part of the model is obtained by

$$ \log \left({\theta}_i\right)=\mu +{\eta}_i+{\phi}_i $$

where *θ*_*i*_ is the relative risk (RR) in neighborhood cluster i, *μ* is an overall mean ratio over neighborhood cluster, *η*_i_ represents specified features of region i which accommodates spatial structure, and *ϕi* denotes unspecified features of neighborhood cluster i which does not incorporate spatial structure. The uncorrelated component *ϕi* is assumed to follow a Gaussian distribution with zero mean and a common variance ${\displaystyle {\sigma}_{\upphi}^2}$

The correlated component *η*_i_ is assumed to follow an intrinsic conditionally autoregressive (ICAR) distribution depending on their neighboring values. In particular,

$$ \eta =\left(\eta 1,\dots, \eta m\right)^{\prime}\sim N\left(0,\varSigma \eta \right), $$

where ${\displaystyle {\sum}_{\eta }={\displaystyle {\sigma}_{\eta}^2}{D}^{-1}},$ and ${\displaystyle {\sigma}_{\eta}^2}$ is the spatial dispersion parameter. The neighborhood matrix D has its i-th diagonal element equal to the number of neighbors of the corresponding region, and the off-diagonal elements in each row equal −1 if the corresponding regions are neighbors and zero otherwise [15,16]. The parameters can be then estimated within the Bayesian framework (MCMC) using non-informative priors for the parameters. This produces the posterior distributions for the parameters in the model. A cluster is defined as an area where the estimated relative risk is significantly larger than 1 (in terms of their credibility sets) [17].

Data manipulation and all statistical analyses were performed using SAS version 9.3. For detecting spatial pattern of hospital readmission, we used WinBUGS software package [18] for all Bayesian analysis to compute the relative risk values. ArcGIS version 10.2.2 (Environmental Systems Research Institute, USA) was used to produce choropleth maps of relative risks. This study was approved by the Health Research Ethics Board at the University of Manitoba (Ethics reference number: H2011:194). Since the data contain personal health information, Health Information Privacy Committee approval to access health administrative data was sought and granted (File number: 2011/2012-13).

## Results

During the four-year study period, 123,842 patients were hospitalized in all Winnipeg hospitals. Of these, 41,551 (33%) were readmitted to hospital in the year following their initial or index discharge. The average age of all patients was 63 years (SD 14). Subjects were 57% female and 43% male. About 70% of patients were married. Among the readmitted patients, the average number of readmissions was 1.94 (SD 1.62) ranging from 1 to 44 readmissions. Most of these readmitted patients (89.4%) had 1or 2 readmissions, while 11.6% of readmitted patients had more than two hospital readmissions after their initial discharge. Table 1 shows the patient characteristics of the study cohort; most of the readmission cases were found to be over 75 years of age. Regarding healthcare utilization and clinical characteristics, readmitted patients had higher rates of not having a regular family physician, CCI at admission > 0, and had an emergent admission at the initial hospitalization.

**Table 1:** **Patient characteristics of all-cause hospital readmission**

**Characteristics**	**Factor**	**Overall (n, %) N = 123,842**	**Readmission times within 12 months (n, %)**		
			**0**	**1-2**	**>2**
			**N = 77,762**	**N = 37,155**	**N = 8,925**
Age	Mean (S.D.)	63.05 (14.07)	61.39 (13.76)	65.4 (14.18)	67.73 (13.89)
	40-51	31,418 (25.37)	22,388 (28.79)	7,591 (20.43)	1,439 (16.12)
	52-62	32,723 (26.42)	21,955 (28.23)	8,912 (23.99)	1,856 (20.8)
	63-74	29,039 (23.45)	17,501 (22.51)	9,191 (24.74)	2,347 (26.3)
	> = 75	30,662 (24.76)	15,918 (20.47)	11,461 (30.85)	3,283 (36.78)
Gender	Male	53,197 (42.96)	32,827 (42.21)	16,010 (43.09)	4,360 (48.85)
	Female	70,645 (57.04)	44,935 (57.79)	21,145 (56.91)	4,565 (51.15)
Marital status	Married	86,769 (70.06)	54,266 (69.78)	26,221 (70.57)	6,282 (70.39)
	Not married/unknown	37,073 (29.94)	23,496 (30.22)	10,934 (29.43)	2,643 (29.61)
Family income	Quintile 1 (most disadvantaged)	23,821 (19.23)	143,81 (18.49)	7,531 (20.27)	1,909 (21.39)
	Quintile 2	22,610 (18.26)	14,001 (18)	7,004 (18.85)	1,605 (17.98)
	Quintile 3	23,724 (19.16)	15,036 (19.34)	6,998 (18.83)	1,690 (18.94)
	Quintile 4	24,752 (19.99)	15,941 (20.5)	7,188 (19.35)	1,623 (18.18)
	Quintile 5 (least disadvantaged)	24,440 (19.73)	15,852 (20.39)	6,953 (18.71)	1,635 (18.32)
	Missing	4,495 (3.63)	2,551 (3.28)	1,481 (3.99)	463 (5.19)
Had a family physician	Yes	44,542 (35.97)	9,010 (33.31)	11,803 (31.77)	1,991 (22.31)
	No	79,300 (64.03)	18,039 (66.69)	25,352 (68.23)	6,934 (77.69)
CCI at admission	0	101,519 (81.97)	66,850 (85.97)	28,905 (77.8)	5,764 (64.58)
	>0	22,323 (18.03)	10,912 (14.03)	8,250 (22.2)	3,161 (35.42)
Emergent admission	Yes	28,292 (22.85)	14,647 (18.84)	10,112 (27.22)	3,533 (39.59)
	No	95,550 (77.15)	63,115 (81.16)	27,043 (72.78)	5,392 (60.41)

**Figure 1:**
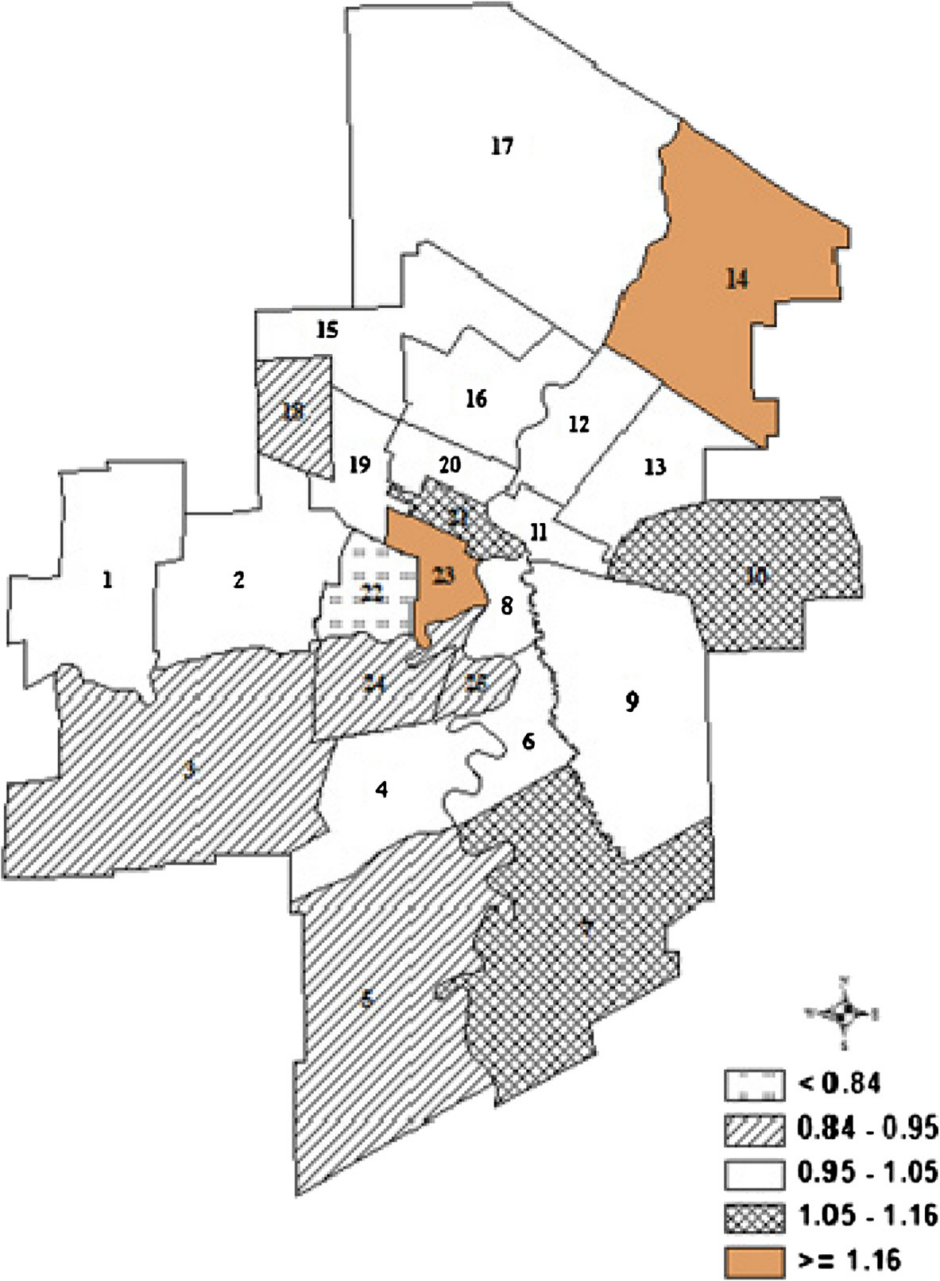
**Smoothed age- and sex- adjusted relative risk rate of readmission in 25 Winnipeg neighborhood clusters.**

Table 2 shows the observed and expected number of cases as well as the relative risk rate of hospital readmission in each neighborhood cluster. We found that there were spatial cluster variations of hospital readmission across the Winnipeg Health Region. The choropleth map depicts the smoothed age- and sex- adjusted relative risk rates of hospital readmission in 25 Winnipeg neighborhood clusters, which ranged between 0.73 and 1.27 readmission during the study period (Figure 1). The highest relative risk rates were observed in the central and northeast sectors of Winnipeg with relative risk rates greater than one (ranging from 1.22 to 1.27). The west and south sectors of Winnipeg had the lowest rates of hospital readmission with relative risk rates less than one (ranging from 0.73 to 0.96). From the BYM analysis, we found that seven neighborhood clusters {1, 7, 9, 10, 14, 21, 23} are more likely to be significant potential clusters for hospital readmissions. Six neighborhood clusters {3, 5, 18, 22, 24, 25} are less likely to be significant potential clusters. These findings suggest that the observed spatial cluster pattern of hospital readmissions in Winnipeg neighborhood clusters is non-random.

**Table 2:** **Relative risk rates of hospital readmissions in 25 Winnipeg neighborhood clusters**

**Neighborhood cluster**	**C**_**i**_	**E**_**i**_	**Relative risk rate**
1	2,477	2,354	1.05*
2	1,968	1,981	0.99
3	2,417	2,547	0.95*
4	1,852	1,916	0.96
5	1,789	2,044	0.88*
6	1,816	1,887	0.97
7	2,311	2,052	1.12*
8	1,146	1,106	1.04
9	2,149	2,041	1.05*
10	2,069	1,792	1.15*
11	898	883	1.02
12	3,160	3,110	1.01
13	1,579	1,522	1.04
14	646	523	1.22*
15	1,211	1,259	0.96
16	2,381	2,301	1.03
17	319	322	1.01
18	724	795	0.92*
19	787	757	1.03
20	1,484	1,471	1.01
21	843	767	1.10*
22	1,751	2,431	0.73*
23	2,214	1,717	1.27*
24	2,184	2,386	0.92*
25	1,255	1,378	0.92*

C _i_ and E_i_ are observed and expected number of cases in neighborhood cluster i.

*p < 0.05.

We also examined the patients’ demographic characteristics such as age and sex in a random-effect Poisson regression model for readmissions; these two factors were not significantly associated with potential cluster of readmissions. However, the result showed that the area-specific random effect parameter (sigma) is 0.23 (*p* < .05), indicating there was spatial variation of readmissions.

## Discussion

There has been increasing concern about rising healthcare costs in Canada. Driven by population aging, the increasing prevalence of chronic diseases, new technologies, inflation and other factors, healthcare costs are anticipated to continue to increase. Healthcare costs are highly skewed across the Winnipeg population owing to a relatively small proportion of persons accounting for a large share of healthcare spending. Since a hospital admission accounts for a large proportion of an individual’s healthcare expenses to the system, the effective preventive efforts aimed at the small number of residents who are at high risk of future readmission to the hospital, could generate net health system savings through averted future hospitalization. Evidence has shown that the interdisciplinary collaborative team approach could reduce hospital readmissions [19,20].

The virtual ward is an innovative model which has been adapted (and, renamed ) for use in the Winnipeg Health Region. One of its principles is to use the daily routines of a hospital ward to provide patients who are recognized to be at high-risk of hospital readmissions by a predictive tool with multispecialty case management at least partially in the home. In the UK, patients who are eligible to be admitted to a virtual ward are identified through the use of a predictive model that helps to identify people who are at high risk of future readmission to the hospital [21]. For example, researchers have developed predictive tools known as PARR (Patients at Risk of Readmission) and Combined model. PARR is a software tool that uses a range of variables from inpatient administrative data sources to calculate the likelihood of hospital readmission over the next 12 months. Unlike PARR, the Combined predictive model is more complex and was designed to produce a more accurate prediction across the whole population, not just persons who have been recently hospitalized [22]. Both predictive models help to identify those individuals most likely to benefit from being admitted to a virtual ward. In the Winnipeg Health Region, we also developed and validated a predictive model using inpatient data. Currently, the work of integrating this predictive model into the Winnipeg Health Region HHTs (virtual wards) is underway.

We believe that our study is the first in Canada to have modeled the relative risk of hospital readmission across a health region by addressing both demographic covariates and spatial effects together. In this study, we examined patients’ sociodemographic, clinical and health related characteristics for all-cause hospital readmission within one year of discharge by using population-based administrative health data. The findings suggest that patients who are older, have a severe comorbidity, have an emergent initial admission, and do not have a regular primary health care provider had higher rates of readmission.

We used the focused spatial statistics technique to test the null hypothesis of no local spatial cluster (pattern) and identify a cluster for a specific region of interest. The results provide a better understanding of the spatial pattern and distribution of hospital readmission in Winnipeg 25 neighborhood clusters. The findings from this study can be used by healthcare services decision makers to plan and target the HHT (virtual ward) services in the high risk areas of the Winnipeg Health Region in order to provide coordinated care to patient in the community after they have been discharged from hospital. In general, we found that the potential clusters of hospital readmissions are located in the central and northeast sectors of Winnipeg. These findings may not reflect the different distributions of other important covariates, such as the primary diagnosis, in these areas that were not measured or adjusted for in our modeling. Further research is necessary to explore these findings.

This study has its strengths. Spatial mapping (Figure 1) depicts a more precise stratification of the risk of readmissions faced by individual neighborhood clusters. We used a Bayesian Poisson model to obtain stable and accurate relative risk rate estimates for all local small neighborhood clusters while retaining geographic and demographic resolution. This study was population-based and represents all hospital readmission cases occurring in the population being studied. We used the Manitoba PHRDR, which is regularly updated and which has comprehensive follow-up data on care. Therefore, these data were found to have high accuracy and quality [23]. This study also has its limitations. First, we only estimated the relative risk rate of all-cause of hospital readmission in this study; additional studies for specific diagnoses such as readmissions related to heart disease or chronic respiratory disease are needed to investigate how the geographic distribution is different amongst the community areas. Second, we were not able to examine the effect of informal care from family members or friends after discharge, which may be associated with high readmission risk, because this information was not available in the administrative data.

## Conclusions

In this study, we identified area variations in hospital readmission rates across the Winnipeg Health Region. Our spatial analysis provided a visual tool to identify spatial pattern or potential spatial pattern of patients who had frequent hospital readmissions. This study provides the implementation guide for a virtual ward type program in the Winnipeg Health Region, which should help to improve long-term chronic condition management in community settings and prevent readmission to hospital.
